# Photosynthesis in a different light: spectro-microscopy for *in vivo* characterization of chloroplasts

**DOI:** 10.3389/fpls.2014.00292

**Published:** 2014-06-30

**Authors:** Sébastien Peter, Martina B. Zell, Christian Blum, Alexander Stuhl, Kirstin Elgass, Marcus Sackrow, Vinod Subramaniam, Alfred J. Meixner, Klaus Harter, Veronica G. Maurino, Frank E. Schleifenbaum

**Affiliations:** ^1^Department of Plant Physiology, Center for Plant Molecular Biology (ZMBP), University of TübingenTübingen, Germany; ^2^Biocenter Cologne, Botanical Institute, University of CologneCologne, Germany; ^3^Nanobiophysics Group and MESA+ Institute for Nanotechnology, University of TwenteEnschede, Netherlands; ^4^Department of Nano Optics, Insitute of Physical and Theoretical Chemistry, University of TübingenTübingen, Germany; ^5^Department of Nanoscale Biophysics, FOM Institute AMOLFAmsterdam, Netherlands; ^6^Plant Molecular Physiology and Biotechnology Group, Institut of Developmental and Molecular Biology of Plants, Cluster of Excellence on Plant Sciences, Heinrich-Heine-UniversitätDüsseldorf, Germany

**Keywords:** fluorescence, spectroscopy, photochemistry, chloroplast, photosystem, spectromicroscopy

## Abstract

During photosynthesis, energy conversion at the two photosystems is controlled by highly complex and dynamic adaptation processes triggered by external factors such as light quality, intensity, and duration, or internal cues such as carbon availability. These dynamics have remained largely concealed so far, because current analytical techniques are based on the investigation of isolated chloroplasts lacking full adaptation ability and are performed at non-physiologically low temperatures. Here, we use non-invasive *in planta* spectro-microscopic approaches to investigate living chloroplasts in their native environment at ambient temperatures. This is a valuable approach to study the complex function of these systems, because an intrinsic property—the fluorescence emission—is exploited and no additional external perturbations are introduced. Our analysis demonstrates a dynamic adjustment of not only the photosystemI/photosystemII (PSI/PSII) intensity ratio in the chloroplasts but also of the capacity of the LHCs for energy transfer in response to environmental and internal cues.

## Introduction

Photosynthesis of plants still sets the benchmark in light-to-energy conversion regarding efficiency, stability and the ability to adapt to dynamic surrounding conditions. Photosynthesis is a highly complex process, involving multi-step energy migration within and between the two photosystems (PS) I and II, which are located in adaptable ratios in the grana and the stroma thylakoids of chloroplasts. The PSs are highly ordered functional and structural units that perform the primary photochemistry of photosynthesis: the absorption of light and the conversion of energy by charge separation. Especially the light harvesting complexes (LHCs) are responsible for the high efficiency of the whole process of photosynthesis due to the broad spectral absorption of ambient light by the LHCs, which is directed to the reaction center via a complex and efficient energy migration chain.

To optimize the use of available light-energy, higher plants undergo highly complex adaptation processes, which are mainly concerned with a structural rearrangement of the chloroplasts (Anderson et al., 1988, 2008; Anderson, 1999). The thylakoid membranes of chloroplasts, which are organized in stacked (grana thylakoids) and non-stacked (stromathylakoids) membranes, comprise different amounts of PSI and PSII. Ultrastructural studies of chloroplasts reveal an accumulation of PSII predominantly in the grana thylakoids, whereas PSI is found mainly in the stroma thylakoids (Anderson, 1986; Allen and Forsberg, 2001). Plants that experience changes in ambient light conditions offer an altered photosynthesis efficiency, which most likely is believed to be connected to a reorganization of the chloroplasts and, hence, a change in the PSI/PSII ratio (Anderson et al., 1988).

Accordingly, it is desirable to determine this ratio *in vivo* at ambient conditions as a measure of the plant ability to adapt its photosynthesic apparatus to the changing light environment. Non-invasive analyses of chloroplasts use the inherent autofluorescence of these compartments, which generally makes them accessible to optical studies. The emission spectrum of chloroplasts exhibits a maximum at 680 nm, which can be assigned to the fluorescence of the reaction center of PSII. Additionally, a spectral shoulder between 700 and 750 nm in the emission spectrum arising from the PSI reaction center is observed (Krause and Weis, 1991). However, a straightforward determination of the PSI/PSII ratio is hampered using conventional fluorescence microscopy due to the strong overlap of the spectral signatures of PSI and PSII. Hence, fluorescence spectroscopic measurements up to now have mostly been carried out at low temperatures (77 K and below) using isolated chloroplasts, where a differentiation between PSI and PSII is possible (Krause and Weis, 1991). However, similar to ultrastructural studies, plant adaptation processes in a living cell context remain obscured with these methods, since the composition of the photosynthetic apparatus reacts highly sensitive to sample treatments (Andersen et al., 1972). Furthermore, the influence of environmental and endogenous cues on the photosynthetic capacity cannot be explored using isolated chloroplasts. Here, we present two strategies using optical spectro-microscopy to overcome these limitations. These non-invasive techniques provide access to the composition of the photosynthesis apparatus and its adaptation to environmental changes at ambient conditions. To this end, we analyzed the PSI/PSII intensity ratio in chloroplasts at room temperature and in their cellular context by applying high spatial resolution fluorescence spectroscopy. We show that in a high-resolution fluorescence emission spectrum, the contributions of the distinct photosystems can be derived from the spectral shape, which depends on the relative contributions of PSI and PSII, respectively, and hence, the two subpopulations can be identified and compared. A careful statistical analysis of the room temperature emission spectra (SART) enables for monitoring global adaptations in the chloroplasts (i.e., the PSI/PS II intensity ratio).

**Figure 1:**
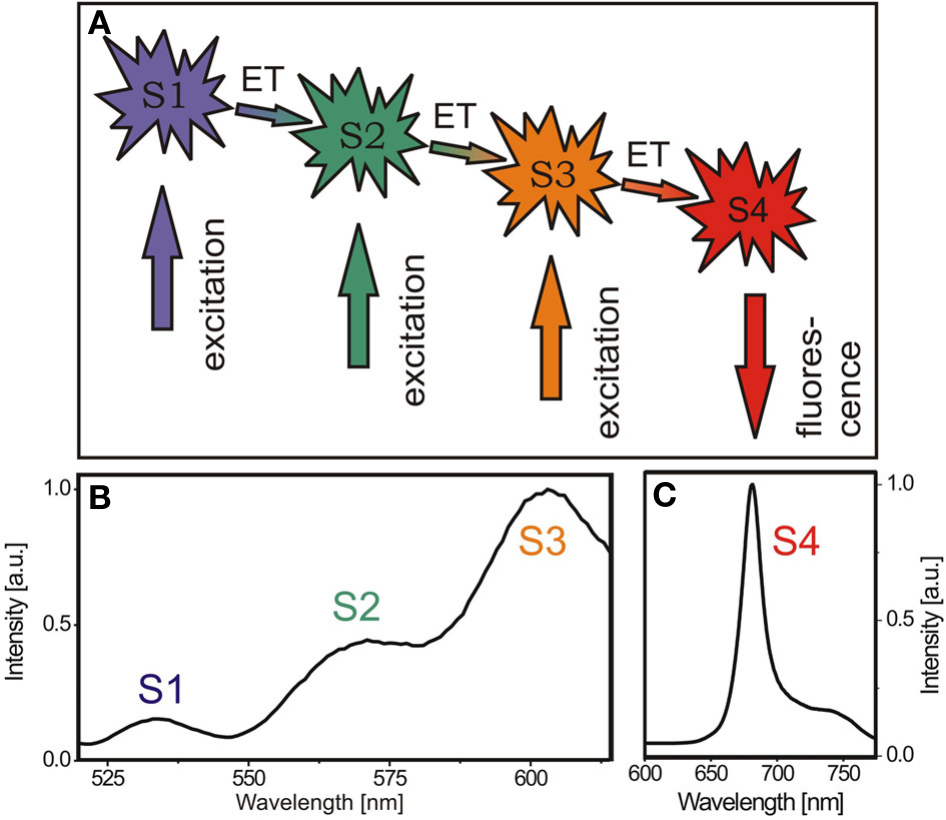
**Energy migration pathway in chloroplasts can be visualized by spatially resolved fluorescence excitation spectroscopy (FExS)**. **(A)** Energy migration scheme in chloroplasts. S1 to S3 indicate states in the LHC energy transfer cascade, from which excitation energy is transferred downstream to the reaction center (S4). **(B)** Representative confocal fluorescence excitation spectrum of an *A. thaliana* chloroplast recorded by sweeping the excitation wavelength while recording the fluorescence intensity at 680 nm. **(C)** Typical chloroplast fluorescence emission spectrum of an *A. thaliana* chloroplast excited at 633 nm.

Using conventional fluorescence-based techniques, however, only the final chromophore of an energy transfer cascade in the photosystems (Figure 1A) can be monitored, while the individual steps and composition of the energy transfer systems are not accessible (Figure 1B). This is because the energy absorbed by the LHCs is very efficiently funneled to chlorophyll *a* in the reaction centers as the last component of the energy migration chain (Butler and Kitajima, 1975) (Figure 1A). Accordingly, any detected fluorescence light originates from the emission of the reaction centers and information about the upstream energy cascade is not available (Figure 1C). The crystal structures of LHCs suggest a rigid, uniform assembly of these complexes (Liu et al., 2004). Variations are known to involve different supramolecular organizations of PSs and LHCs detected by electron microscopy (Yakushevska et al., 2001) as well as alterations in the total pigment amount and ratio in the chloroplasts (Bailey et al., 2004). Pigment analysis is usually based on the isolation of pigments using organic solvents followed by chromatographic separation. However, a technique that enables the observation of the photosynthetic efficiency *in vivo* has been missing so far. To overcome this restriction and get access to the composition of the energy transfer complexes *in vivo*, we established a novel *in planta* approach by disentangling the distinct energy transfer steps at ambient temperature. This was achieved by recording the fluorescence excitation spectra (FExS) in the diffraction limited focal spot by confocal fluorescence microscopy.

## Methods

### Plant material and growth conditions

Arabidopsis (*Arabidopsis thaliana*) ecotype Columbia-0 (wild type, wt) and transgenic plants overexpressing the maize photosynthetic NADP-malic enzyme (MEm2 and MEm5 Fahnenstich et al., 2007) were grown in pots containing three parts of soil (Gebr. Patzer KG, Sinntal-Jossa, Germany) and one part of vermiculite (Basalt Feuerfest, Linz, Germany) at 22°C day temperature under long days (LD, 16 h light/8 h darkness) or short days (SD, 8 h light/16 h darkness). The light intensity (photosynthetic active radiation) was 110 μmol quanta m^−2^s^−1^. Maize plants were grown in soil with temperatures ranging from 23 to 26°C and a 16 h light/8 h darkness cycle at a photon flux density of 350 μmol quanta m^−2^s^−1^. Mature (12 week-old plants) and immature (2 week-old seedlings) maize plants were used for the measurements.

### Statistical analysis of room temperature emission spectra (SART)

Data acquisition for SART was accomplished by initially recording a fluorescence intensity map of the plant tissue to identify the chloroplasts using a custom-built confocal fluorescence microscope based on a Zeiss Axiovert 135TV (Carl Zeiss, Germany, Detector: PDM avalanche photodiode, Picoquant, Germany). The custom-built setup was equipped with a 633 nm excitation source (PL-610, Polytec, Germany) and a raster scanning stage (P517, Physik instrumente, Germany). Fluorescence emission spectra with diffraction-limited spatial resolution were acquired from chloroplasts to determine the relative contributions of PSI and PSII to the spectral signature using a polychromator spectrograph (Acton 300i, Roper Scientific, US, Pixis 100B, Princeton Instruments, US). To reduce the data volume, this spectral shape was analyzed by studying the intensity ratio at characteristic wavelengths. The PSI to PSII intensity ratio was directly deduced by calculating the intensity ratio λ_emPSI(730 nm)_:λ_emPSII(680 nm)_. To account for individual differences in the composition within and among single chloroplasts, we recorded five spectra per chloroplast and averaged the PSI/PSII intensity ratio of numerous chloroplasts (*n* = 140–200). Based on this statistical analysis of chloroplast emission spectra at room temperature (SART), the average PSI/PSII intensity ratio of living plants was determined as a function of external conditions.

### Fluorescence excitation spectro-microscopy (FExS)

To record fluorescence excitation spectra with a diffraction limited spatial resolution, our custom-built confocal microscope (details above) was combined with a supercontinuum laser (SC-400pp, Fianium, UK) as an excitation source. To sequentially scan the excitation wavelength, the supercontinuum source was coupled into a grating monochromator (Acton 300i, Roper Scientific, US), which was synchronized with the data acquisition by a custom-built software protocol. The fluorescence emission was recorded in a spectral window between 655 and 685 nm, matching the fluorescence emission of the PS II reaction center. Excitation spectra were recorded by sweeping the excitation wavelength from 500 to 620 nm, covering the absorption of the LHC pigments while recording the fluorescence emission. Using this fluorescence excitation spectroscopy (FExS) arrangement, intense fluorescence emission is observed for excitation waengths in resonance with the absorbance of one of the LHC pigments. Simultaneously, the energy migration pathway is intact so that the excitation energy is funneled downstream to the reaction center. Accordingly, the intensity of the distinct bands is proportional to the relative amount of energy guided to the reaction center (Figure 1). A detailed description of the experimental realization by the combination of confocal microscopy with a feedback-controlled supercontinuum laser source is given elsewhere (Blum et al., 2011).

## Results

### Using statistical analysis of room temperature emission spectra (SART) to monitor the PSI/PSII intensity ratio of individual chloroplasts

**Figure 2:**
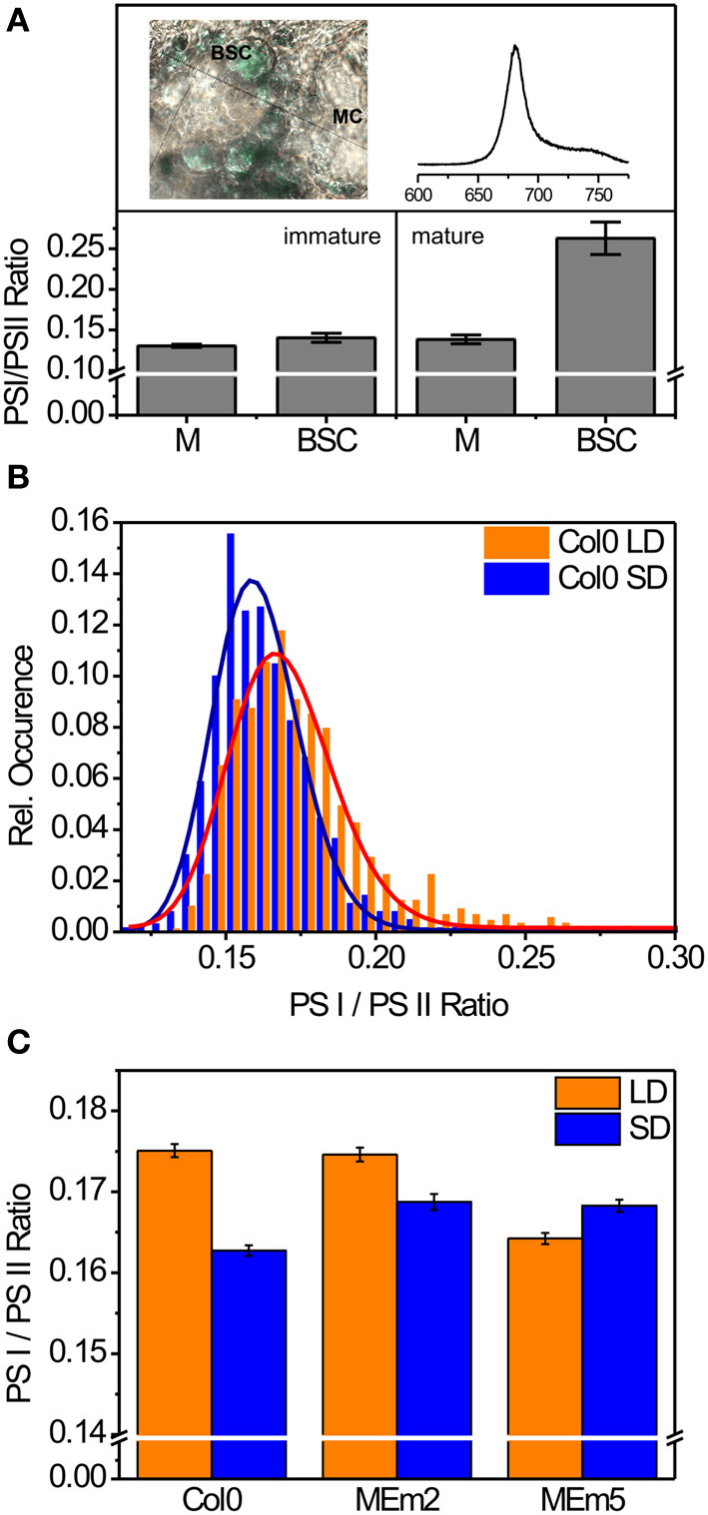
**PSI/PSII ratio in chloroplasts of living plant cells determined by SART**. **(A)** Average PSI/PSII ratio of chloroplasts in immature maize leaf tissue [left; calculated from 19 spectra recorded from mesophyll cells (MC) and 19 spectra recorded in bundle sheath cells (BSC), (*n* = 38, *p* = 0.1)] and in mature leaf tissue (right; calculated from 38 spectra recorded in MC and 59 spectra recorded in BSC). The upper left shows a brightfield image of a maize leaf section with MS and BSC cells. The upper right depicts a typical fluorescence intensity spectrum recorded from MC (x axis: emission wavelength [nm]). **(B)** Histogram of the calculated PSI/PSII intensity ratio of *A. thaliana* grown under long day (LD) and short day (SD) conditions. The histograms were calculated from 893 spectra in case of LD plants and 630 spectra in case of SD conditions. The histograms were fit with lognormal model functions with an adjusted *R*^2^-value of at least 0.95 (solid lines). **(C)** Average PSI/PSII intensity ratios in chloroplasts of *A. thaliana* wild type (Col0) and transgenic plants expressing maize plastidic NADP malic enzyme at different levels (MEm2 and MEm5 plants with 6 and 33 times increased NADP ME activity with regards to the wild type, respectively) grown under short day (SD) or long day (LD) conditions.

At room temperature, the emission spectra of PSI and PSII show significant spectral overlap and cannot be distinguished using band-pass filtering as a straightforward approach. However, in a high-resolution fluorescence emission spectrum, the obtained spectral shape depends on the relative contributions of PSI and PSII, and hence the two subpopulations can be identified, compared, and distinguished from each other (Figure 2A). To survey the suitability of the SART approach for PSI/PSII intensity ratio determination, chloroplasts of immature and mature leaves of maize (*Zea mays*), a canonical C4 plant with Kranz anatomy consisting of mesophyll and bundle sheath cells, were used as model system. Maize chloroplasts of immature bundle sheath cells exhibit a high PSII activity (Lozier et al., 1971) while those of mature bundle sheath cells have established the characteristic agranal or only rudimentary grana morphology and show a low PSII activity (Woo et al., 1970). Using the *in vivo* SART approach we determined a low PSI/PSII ratio in bundle sheath cells of immature maize leaves, whereas this ratio rose substantially for bundle sheath cells of mature plants (Figure 2A). Based on this data, we validated that SART enables the determination of the PSI/PSII ratio in living plants. We therefore used SART to investigate the ability of the prototypical C3 plant *Arabidopsis thaliana* (Arabidopsis; *A. thaliana*) to adjust the PSI/PSII ratio to external and environmental cues.

To check the influence of the duration of the light period on the relative PSI/PSII luminescence, we grew *A.thaliana* ecotype Columbia-0 (Col0) under long day (LD) and short day (SD) conditions (16/8 and 8/16 h photoperiod, respectively). Spectra were recorded from plants at the same growth stadium in the second half of the light period. To prevent any adulteration of the results by spectra close to the noise or saturation limit of the spectrograph, we disregarded the most and least intense 5% of the recorded spectra for downstream processing. The calculated PSI/PSII ratios show a monomodal distribution that can be well explained by a lognormal model in both LD- and SD-grown plants (Figure 2B). As a *t*-test analysis might be misleading to identify whether the means of these distributions differ significantly, we performed a Z-score test using unbiased estimators (Zhou et al., 1997) to calculate the significance level for this lognormal distributions according to

α=2φ(Z); Z<0

with

$$\varphi \left(Z\right)=\frac{1}{\sqrt{2\pi }}\int_{-\infty }^{Z}exp\left(\frac{-x^{2}}{2}\right)dx$$

Plants grown under LD conditions show an overall significantly increased PSI/PSII ratio compared to plants grown under the more restrictive SD conditions. We calculated a Z-Score of *Z* = −12.57 translating into a significance level of 2.9^*^10^−36^. These results indicate that *A. thaliana* plants adapt their photosynthesis apparatus, namely the apparent PSI/PSII ratio, depending on the available amount of light.

To investigate the capability of the *in vivo* adjustment to changes in internal cues, we compared the PSI/PSII ratio of *A. thaliana* wild type plants with that of two independent transgenic lines overexpressing the maize photosynthetic NADP-malic enzyme (MEm2 and MEm5 with 6 and 33 times higher NADP-ME activity Fahnenstich et al., 2007). As shown previously, the MEm plants present a sustained low level of the organic acids malate and fumarate, especially when grown in SD (Fahnenstich et al., 2007; Zell et al., 2010). As a consequence of the impairment in the supply of carbon in SD, the MEm plants show a dwarf growth phenotype, which is accompanied by more crowded thylakoid membrane systems and a lower PSII quantum yield compared to the wild type. The intensity of the changes described follows a dose response relationship with the level of NADP-ME activity (Zell et al., 2010). However, no differences in phenotype and all the above-mentioned parameters were found when the MEm plants were grown under LD conditions (Zell et al., 2010). For the determination of the PSI/PSII intensity ratio by SART, we grew the plants at both LD and SD conditions and recorded several hundred emission spectra per condition and plant. Again, the histograms of the calculated PSI/PSII intensity ratios obtained are monomodal and plausibly explained by a lognormal model (Supplemental Figure S1). Comparing the means of the PSI/PSII distributions, we found that MEm2 plants show no significant change in their PSI/PSII intensity ratio compared to wild type plants under LD conditions (significance level: 0.64). Furthermore, the MEm2 plants have a moderately enhanced PSI/PSII intensity ratio compared to wild type plants under SD conditions (significance level: 6.5^*^10^−8^). This indicates that the slight overexpression of the plastidic NADP-malic enzyme has, if at all, only a moderate effect on the PSI/PSII intensity ratio in SD conditions. In contrast, the MEm5 plants show a pronounced drop in the PSI/PSII intensity ratio under LD conditions and slight increase under SD conditions (Figure 2C; significance level of 5.3^*^10^−27^ and 5.8^*^10^−9^ at LD and SD, respectively). This indicates that the chloroplasts of the MEm5 plants adapt to the carbon shortage by lowering the PSI/PSII intensity ratio under LD conditions. Since the quantum yield of PSII does not depend on the NADP-malic enzyme expression level when grown under LD conditions (Zell et al., 2010), this change in the PSI/PSII intensity ratio reflects an alteration in the relative PSI amount. Possibly, the alteration of the PSI/PSII intensity ratio in LD is one compensatory mechanism that enables the MEm5 plants to prevent a dwarfed growth phenotype.

### Using fluorescence excitation spectro-microscopy (FExS) to investigate the efficiency of light harvesting complexes in an individual chloroplast

**Figure 3:**
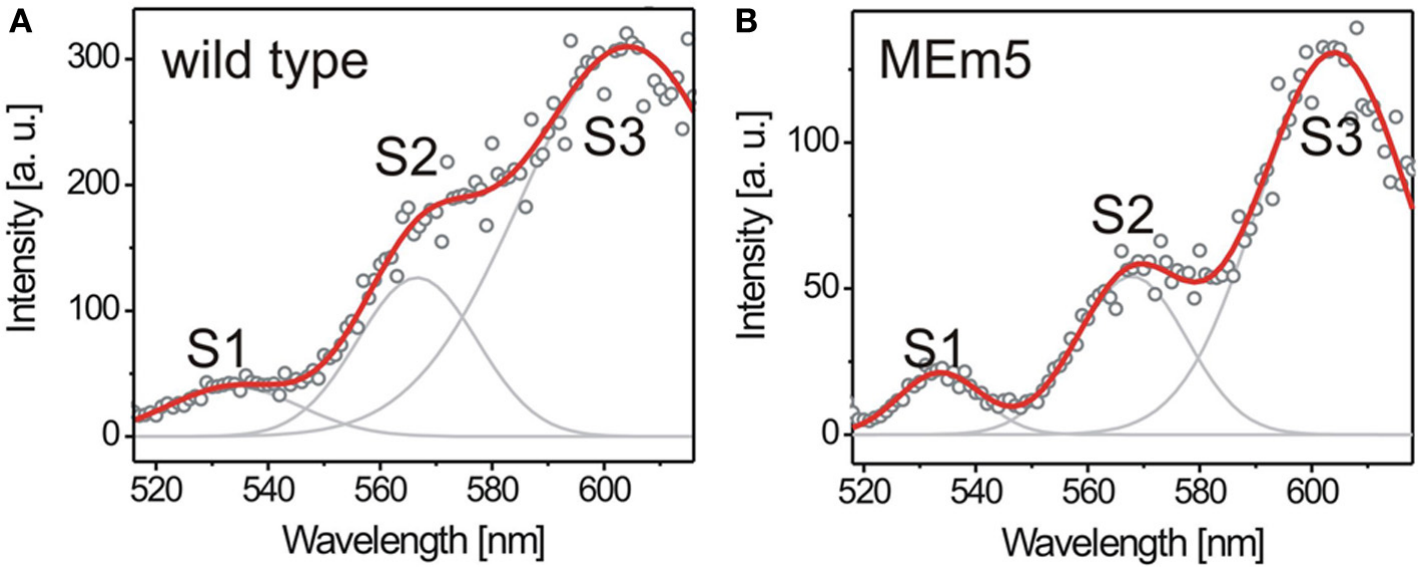
**Fluorescence excitation spectra of chloroplasts recorded from 5-week-old *A. thaliana* wild type (100 spectra) (A) and MEm5 (150 spectra) (B)**. The plants were grown at white light (50 μmol quanta m^2^s^−1^) under SD conditions. Three transitions contributing to the energy migration chain are visible as distinct bands. The spectral envelope was fitted by three Gaussians (gray lines) to determine the relative contribution of the respective pigments to the excitation spectrum. The red curve depicts the summation of the three Gaussian distributions; round circles represent the raw data. Fluorescence intensity at the red region of the spectrum is slightly overrepresented due to higher excitation power at that edge of the spectrum.

To investigate the individual efficiency of LHCs and their ability to adapt to changing environmental conditions, we applied FExS to 5-weeks-old *A. thaliana* wild type and MEm5 plants grown under SD conditions. The emission passband was chosen to match the PSII-emission. As shown in Figure 3, wild type and MEm5 plants showed three fluorescence excitation peaks between 520 nm and 620 nm (λ_peak1_ = 534 nm; λ_peak2_ = 568 nm; λ_peak3_ = 604 nm). We initially assumed these three peaks to originate in different pigments; however, there are no three pigments exhibiting their absorbance maxima in this wavelength regime. Rather, the peaks are assigned to the Q_x_(0, 0) and higher vibronic Q_x_ and Q_y_ transitions of the chlorophylls (Breton and Vermeglio, 1988; Fragata et al., 1988). The excitation spectra shown here reflect how much light is funneled to the PSII reaction centers upon absorbance at a given wavelength. Assuming that the LHCs and PSs form highly optimized functional units one expects that there is little variation in the composition of these cellular compounds and, as a consequence, this is expected to be reflected in a very constant excitation spectrum.

To establish if there is any adaptation of plants not only in the PIR but also inside the light harvesting apparatus, we recorded chloroplast excitation spectra *in planta* from wild type and from MEm5 plants. For data analysis, three Gaussians were fit to every spectrum as shown in Figure 3 and the areas under the peaks were calculated in relation to the area under the first peak at 534 nm (peak S1).

**Figure 4:**
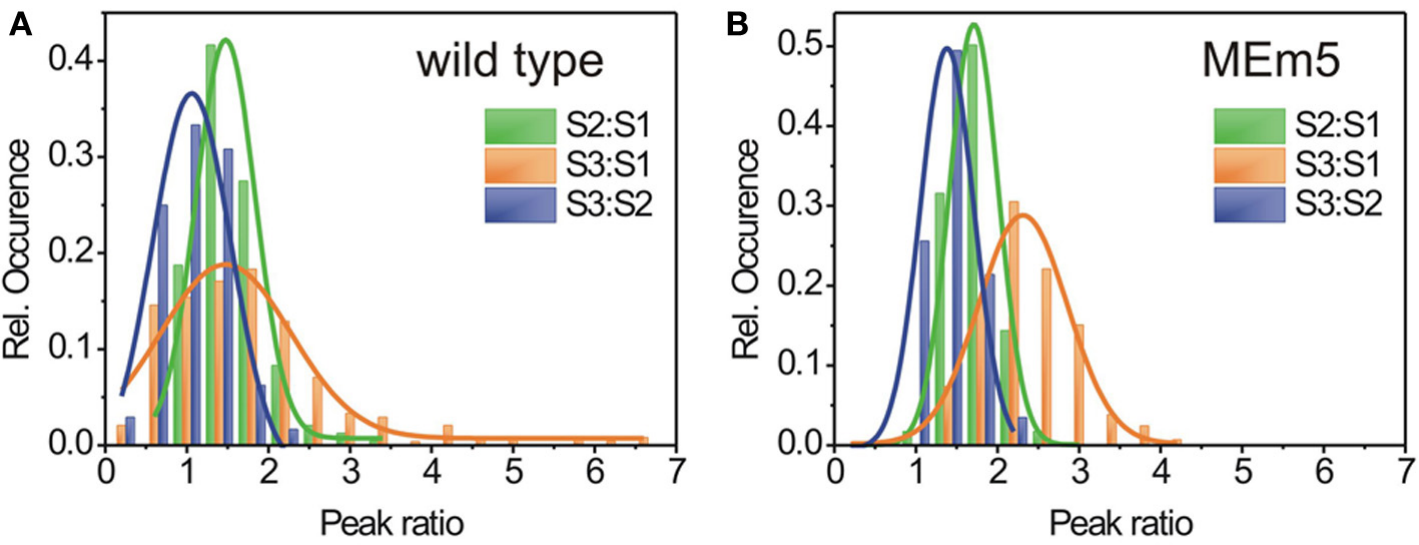
**FExS reveals characteristic composition for different carotenoids of the LHCs in chloroplasts of *A. thaliana* wild type (A) and MEm (B) plants**. Every FExS spectrum was fitted by three Gaussians and the area under the peaks was calculated relative to the peak representing the highest transition energy (S1). Histograms of the peak ratio distribution of the excitation peak areas for wild type **(A)** and MEm5 plants **(B)**. Red: peak ratio S2:S1, green: peak ratio S3:S1; blue: peak ratio S3:S2. The histograms were fit by Gaussians (solid lines) and show normal distribution.

As shown in Figure 4A, the spectra of wild type plants showed a rather broad distribution of the peak area ratios [2σ_(S2:S1)_ = 0.73 ± 0.05, σ_(S3:S1)_ = 1.60 ± 0.14, 2σ_(S3:S2)_ = 0.96 ± 0.24). This indicates that light harvesting in living plants is by no means due to a rigid, uniform machinery but rather exhibits variations in terms of efficiency on a local basis. In contrast, for the MEm5 plants, the peak ratio distributions were significantly narrowed compared to the wild type [2σ_(S2:S1)_ = 0.60 ± 0.01, σ_(S3:S1)_ = 1.09 ± 0.08, 2σ_(S3:S2)_ = 0.65± 0.04) (Figure 4B). Furthermore, the peak ratios exhibit an overall shift toward larger values for MEm5 plants compared to their wt counterparts. This means that overall, a higher fraction of fluorescence is excited at higher wavelength regions in MEm5 plants than in Col0.

## Discussion

The results reported in this manuscript offer several insights into the adaptation potential of the photosynthetic apparatus of higher plants. First, our SART data substantiate previous findings that differences in the length of the light period in live plants lead to a reprogramming of the PSI/PSII ratio. We believe that the measured change in the PSI/PSII intensity ratio of LD- and SD-grown plants can be translated into a readjustment in the PSI/PSII ratio in plants because the quantum yield of Col0 plants grown in the applied light conditions does not change significantly (Zell et al., 2010). In contrast to previous studies, our data were obtained applying a non-invasive approach at ambient temperature conditions. As our data support the results of ultrastructural experiments, we consider our technique valid to monitor the PSI/PSII intensity ratio in living plant cells. We showed that SART can be used to investigate adaptation processes in chloroplasts of plants with changes in carbon homoeostasis with high sensitivity.

In LD conditions, MEm2 plants show no distinguishable growth and PSI/PSII intensity ratio phenotype from wild type plants, although they exhibit slightly lower levels of CO_2_ assimilation (Zell et al., 2010). Under this photoperiod, it appears that the photosynthetic productivity can still compensate for the physiological carbon depletion. Therefore, a significant adjustment of the PSI/PSII intensity ratio in MEm2 and wild type plants grown under LD conditions is not required. Contrarily, MEm5 plants, which exhibit a much higher level of NADP-ME, show clear changes in the PSI/PSII intensity ratio under LD conditions. Apparently, the carbon deficit under these conditions is already severe enough to provoke an effect in the composition of the photosynthetic apparatus. Interestingly, other parameters related to photosynthesis such as the total chlorophyll content and the PSII quantum yield are not different between MEm5 and wild type plants (Zell et al., 2010). Under SD conditions, there is a clear growth phenotype for both MEm2 and MEm5 plants. This is accompanied by a change in the PSI/PSII intensity ratio in MEm plants compared to wild type. MEm plants grown under SD conditions furthermore experience a decreased PSII quantum yield compared to wt plants which is progressive with the NADP-ME activity (Zell et al., 2010). Interestingly, the difference in the PSI/PSII intensity ratio of MEm2 and MEm5 plants is not linear with the NADP-ME activity. We observed that the PSI/PSII intensity ratio of MEm2 plants decreases from LD- to SD-grown plants whereas in MEm5 plants there is an increase. The altered PSII quantum yield might well affect the measured PSI/PSII intensity ratio. However, the fact that these two effects are not correlated is a strong indication that the observed change in the PSI/PSII intensity ratio originates in a relative amount of PSI/PSII. This finding is interpreted as follows: Under sufficient light conditions—as is the case in LD—there is enough energy supply to compensate for the carbon deficit allowing normal growth. In case of MEm2, there is no adaptation necessary due to the moderate overexpression of the NADP-ME. In MEm5, however, a detrimental growth phenotype may be avoided by physiological/molecular compensatory changes, being one of them the large reorganization of the photosynthetic apparatus. In SD conditions, the photosynthetic productivity of MEm chloroplasts can no longer overcome the reduced CO_2_ assimilation due to a large carbon starvation. Even a possible re-adjustment of the PSI/PSII ratio, which might reflect a general, adaptive response to limiting light conditions, cannot compensate for the carbon deficit.

A deeper insight into the LHCs of chloroplasts is obtained by application of the FExS approach, which allows identifying the direct observation of the amount of light that is funneled to the downstream excitation centers after excitation of a specific energy state. Our FExS data revealed that the energy transfer to the PSII reaction centers upon excitation in different wavelength regimes is not constant but exhibits significant variations within a plant. The three excitation peaks described in this study do not translate into the amounts of different pigments involved in light harvesting. Rather, we interpret the energy cascade in LHCs as complex multi-level entities. If the LHCs were uniformly assembled, no variations at all would be observed in the excitation peak ratios, just like in the excitation spectra of fluorophores in solution. The fact that we observe strong variations here means that the efficiency of downstream energy transfer upon excitation of a distinct energy level is not constant. As a consequence, a varying fraction of the collected light might migrate by pathways different than energy transfer to the PSII reaction center upon excitation, arguably by non-radiative deactivation of the excited state. The fluctuations observed here essentially reflect a different nano-environment of the excited pigments not observable in isolated systems. This indicates that the chlorophylls of the energy cascade involved in light harvesting are not rigidly embedded into the protein scaffold but rather represent a highly dynamic system *in vivo*. Interestingly, we found that MEm5 plants suffering from carbon starvation exhibit an overall altered photosynthetic efficiency in the probed spectral regime *in vivo* along with a narrowed distribution of the excitation peak ratios. This indicates an overall altered, more uniform composition of MEm5 chloroplasts compared to wild type plants. On microscopic level, wild-type plants seemingly maximize the light absorption and the subsequent energy transfer to the reaction centers. The MEm5 plants obviously present a limitation of this ability. Likely, the MEm5 plants are not able to dynamically re-organize their LHCs like the wild type due to the lack of sufficient carbon supply during the extended night period (Zell et al., 2010). This may reduce the capacity of the MEm5 plants to optimize the photosynthetic energy generation process. Together with the de-regulation of the PSI/PSII ratio, this could contribute to the carbon starvation phenotype of the MEm5 plants under SD conditions.

Taken together, our results suggest the potential for adaptations within the LHCs of land plants such the *A. thaliana* based on a different interaction network of the pigments involved in light harvesting, a mechanism differing from the chromatic adaptation previously reported to be due to alterations of the overall LHC pigment composition incyanobacteria (Kehoe and Gutu, 2006; Mascle-Allemand et al., 2010).

## Concluding remarks

In this work, we presented two spectro-microscopic approaches, namely SART and FExS, to investigate for the first time photosynthetic adaptation processes in single chloroplasts of living plant cells in their native milieu at ambient temperature. Our data show that SART is a sensitive method for detecting and quantifying externally- (light conditions) and internally- (carbon homoeostasis changes) regulated adaptation processes of chloroplasts. This method is highly sensitive and is thus well suited to investigate the dynamics of PSI/PSII ratio of chloroplasts *in vivo*. Whereas SART spectroscopy enables the determination of the relative PSI/PSII amounts, FExS allows the disentanglement of the energy transfer processes in the LHCs. Both the PSI/PSII fluorescence intensity ratio and the energy transfer are demonstrated to be adjustable to external conditions such as light intensity and internal to cues, such as C_4_ acid deficiency, in a highly flexible manner.

## Conflict of interest statement

The authors declare that the research was conducted in the absence of any commercial or financial relationships that could be construed as a potential conflict of interest.
