# Supplementary material for: Photosynthesis in a different light: spectro-microscopy for in vivo characterization of chloroplasts
Source: Front Plant Sci. 2014 Jun 30;5:292. doi: 10.3389/fpls.2014.00292 (PMC4082301; doi:10.3389/fpls.2014.00292)
Supplement: Supplementary file 1 [file DataSheet1.DOCX]

**Supplementary Information**

Figure 1

Supplementary Figure 1: Histograms of measured PSI/PSII emission ratios for MEm2 (top panel) and MEm5 (bottom) plants grown under long day (LD, orange bars) and short day (SD, blue bars) conditions along with lognormal fits (solid lines). Spectra were recorded from plants in identical physiological states. To prevent any adulteration of the results by spectra close to the noise or saturation limit of the spectrograph, the 5% of the spectra with the highest and lowest PSII intensity were disregarded for further processing. The histograms were calculated from 885 (MEm2 LD), 810 (MEm2 SD), 888 (MEm5 LD) and 896 spectra (MEm5 SD). The lognormal fits yielded an adjusted R^2^ value of at least 0.95.
